# Impact of Aerobic Training on Transcriptomic Changes in Skeletal Muscle of Rats with Cardiac Cachexia

**DOI:** 10.3390/ijms26136525

**Published:** 2025-07-07

**Authors:** Daniela Sayuri Inoue, Quinten W. Pigg, Dillon R. Harris, Dongmei Zhang, Devon J. Boland, Mariana Janini Gomes

**Affiliations:** 1 Department of Kinesiology and Sport Management, Texas A&M University, College Station, TX 77845, USA; dani.yoshimura_7@tamu.edu (D.S.I.); qwpigg99@tamu.edu (Q.W.P.); dillon_harris@tamu.edu (D.R.H.); 2 Texas A&M Institute for Genome Sciences & Society, College Station, TX 77843, USA; dongmei@tamu.edu (D.Z.); devonjboland@tamu.edu (D.J.B.); 3 Department of Molecular Pathogenesis and Immunology, Texas A&M University, College Station, TX 77807, USA

**Keywords:** pulmonary arterial hypertension, exercise, metabolism, muscular remodeling, transcriptome

## Abstract

Cardiac cachexia (CC) is an advanced stage of heart failure (HF) characterized by structural and functional abnormalities in skeletal muscle, leading to muscle loss. Aerobic training provides benefits; however, the underlying molecular mechanisms remain poorly understood. This study aimed to investigate the therapeutic effects of aerobic training on transcriptomic alterations associated with disease progression in cachectic skeletal muscle. HF was induced in male Wistar rats by a single monocrotaline injection (60 mg/Kg). Aerobic training consisted of 30 min treadmill running at ~55% of maximal capacity, 5×/week for 4 weeks. Assessments included body mass, right ventricle mass, skeletal muscle fiber size and exercise tolerance. RNA-seq analysis was performed on the medial gastrocnemius muscle. Sedentary cachectic rats exhibited 114 differentially expressed genes (DEGs) while exercised cachectic rats had only 18 DEGs. Enrichment pathways analyses and weighted gene co-expression network analysis (WGCNA) identified potential key genes involved in disrupted lipid metabolism in sedentary cachectic rats, which were not observed in the exercised cachectic rats. Validation of DEGs related to lipid metabolism confirmed that *Dgat2* gene expression was modulated by aerobic training in CC rats. These findings suggest that aerobic training mitigates transcriptional alterations related to lipid metabolism in rats with CC, highlighting its therapeutic potential.

## 1. Introduction

Abnormalities in skeletal muscle structure as well as function are common in several diseases, including chronic heart failure (HF). However, in the advanced stages of HF, excessive muscle wasting and concomitant strength loss—referred to as cardiac cachexia (CC)—can occur, affecting up to 15% of HF patients [1]. CC is associated with a significant decline in quality of life and a high annual mortality rate of 20–40% [2]. Given the persistently high number of HF cases [3] and projections estimating the prevalence of HF to reach 11.4 million by 2050 [4], improving the prognosis and treatment for CC is imperative.

Exercise intolerance is a recognized symptom of CC [1], and the underlying pathophysiological mechanisms include inflammation, hypoxemia and both metabolic and mitochondrial dysfunction in skeletal muscle [5,6]. Treatment for CC typically involves a multidisciplinary approach, with regular aerobic training as a core therapeutic prescription [7]. Both the American Heart Association and the American College of Cardiology recognize that exercise training can produce greater benefits in physical capacity than some pharmacological strategies for HF treatment [8]. More specifically, aerobic training is touted to offer a range of benefits, including anti-inflammatory, cardiovascular, and antioxidant effects, as well as enhanced metabolic responses which may be especially pronounced in skeletal muscle [9]. This heightened response is likely due to the greater plasticity of skeletal muscle compared to cardiac muscle [8,9] highlighting the critical role of exercise training in mitigating the symptoms of CC.

Despite current treatment strategies, the effectiveness of interventions for CC remains limited, and further improvement is needed to reduce its prevalence, lower mortality risk, and enhance patients’ quality of life. As a complex and multifaceted disease [10], many of the molecular mechanisms underlying CC remain unclear or unknown, posing a challenge to the development of more effective therapies. Alterations at the gene expression level may provide valuable insights into skeletal muscle remodeling as a consequence of HF [11,12]. For example, Moraes et al. [12] reported altered expression of genes related to muscle fiber regeneration and extracellular matrix remodeling in predominantly oxidative muscle fibers of rats with pulmonary arterial hypertension. Similarly, Yegorova et al. [11] identified gene network alterations related to muscle structure and energy metabolism in the diaphragm of rats with myocardial infarction.

CC and its impact on skeletal muscle have been extensively studied, specifically in the context of exercise interventions. Prior research has established the general benefits of aerobic training on muscle preservation and functional outcomes in HF models [9,13]. However, the molecular mechanisms—particularly at the transcriptomic level—remain poorly defined. Our study addresses this gap by combining functional and morphological assessments with comprehensive RNA sequencing and bioinformatics to identify key transcriptional changes in response to disease and exercise. The purpose of this study was to investigate the therapeutic effects of aerobic training on transcriptomic alterations associated with disease progression in cachectic skeletal muscle. We hypothesized that aerobic training would mitigate the HF-induced transcriptional alterations linked to CC. By investigating training-responsive targets, our study provides new insights into molecular mechanisms that may underlie the therapeutic benefits of exercise in CC.

Our main findings indicate that aerobic training has a protective effect at the transcriptional level. This effect appears to be associated with the expression of key hub genes related to muscle remodeling, lipid metabolism, and mitochondrial function, which were genes found altered in the untrained condition only. While the benefits of aerobic training as part of HF interventions are well documented, the novelty of this study lies in identifying potential new target genes and signaling pathways involved in the effects of aerobic training on CC. Our results lay the groundwork for future studies aimed at elucidating the underlying mechanisms and validating these findings at the protein level to improve both non-pharmacological and pharmacological therapies for CC. Moreover, comparative research examining alternative training protocols and durations may further optimize exercise-based interventions for this condition.

## 2. Results

### 2.1. Characterization of Right Ventricle Remodeling and Cardiac Cachexia Indicators

**Figure 1:**
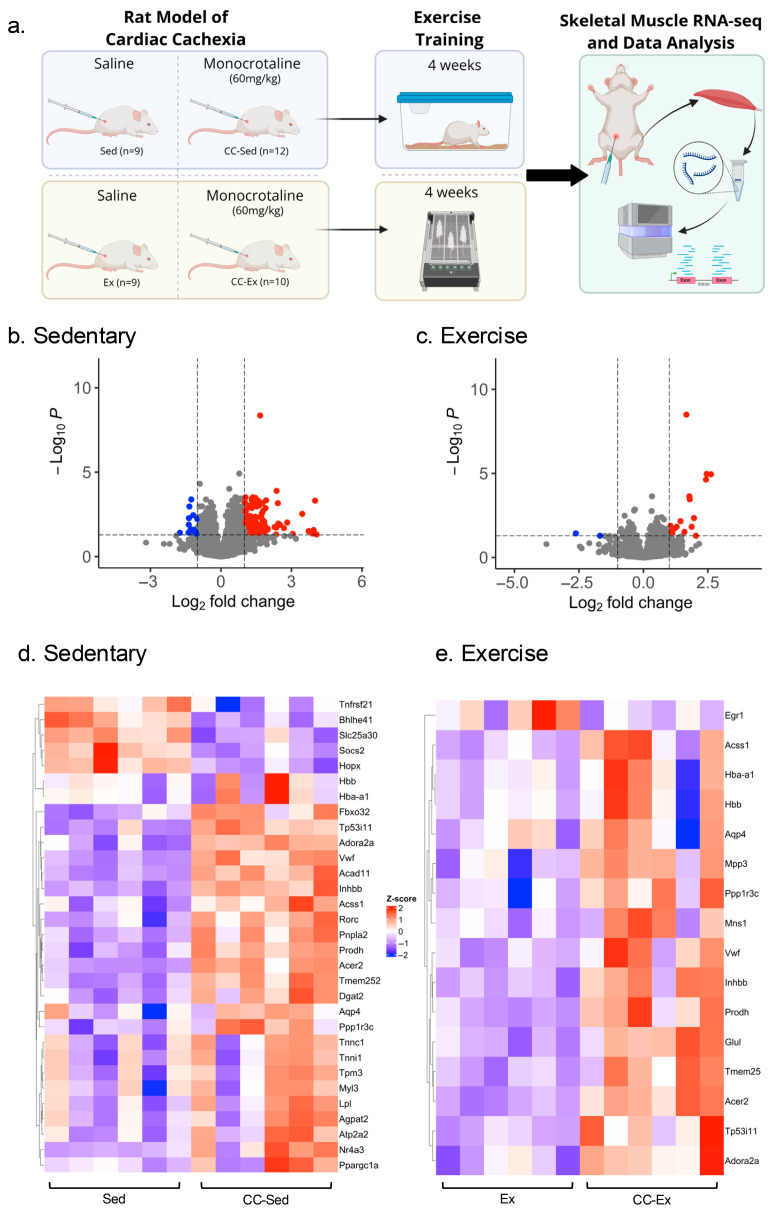
Summary of experimental design and representation of differentially expressed genes (DEG). (**a**) Animal model of cachexia, exercise intervention and skeletal muscle RNA-sequence analysis. Volcano Plots show DEGs based on their log fold change for the (**b**) sedentary (Sed vs. CC-Sed), and (**c**) exercise (Ex vs. CC-Ex); red dots are upregulated DEGs, blue dots are downregulated DEGs, and grey dots are non-significant DEGs. Heat maps represent some DEGs based on z-score for the (**d**) sedentary and (**e**) exercise comparisons. Sed: control sedentary; Ex: control exercised; CC-Sed: cardiac cachexia sedentary; CC-Ex: cardiac cachexia exercised.

**Figure 2:**
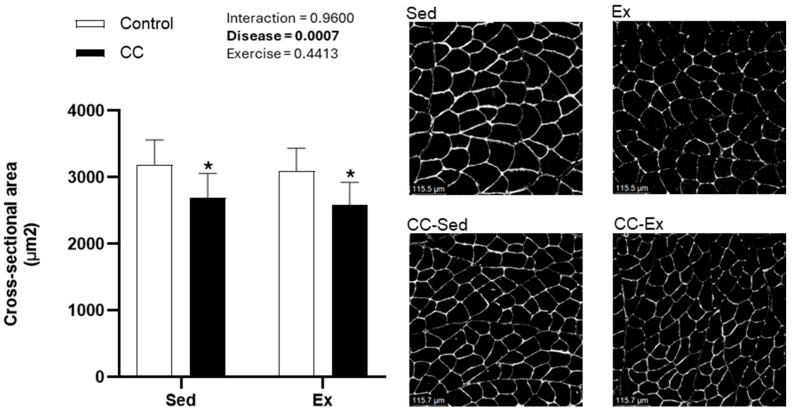
Skeletal muscle histology. Left: Medial gastrocnemius cross-sectional area. Right: Representative gastrocnemius muscle sections. Laminin (white) was used as a membrane marker. Objective 20x. Sed: control sedentary group (*n* = 9); Ex: control exercised (*n* = 8); CC-Sed cardiac cachexia sedentary group (*n* = 7); CC-Ex: cardiac cachexia exercised group (*n* = 6). * Main effect of Disease.

Figure 1a summarizes the experimental design. At the end of the experiment, the control sedentary group (Sed) contained all initial nine animals, while one animal from the control exercise group (Ex) was excluded due to refusal to exercise, resulting in eight animals. In the cardiac cachexia sedentary group (CC-Sed), 4 animals died during the experiment, and 1 was excluded post-mortem for not exhibiting right ventricle (RV) remodeling. Similarly, three animals in the cardiac cachexia exercise group (CC-Ex) died, and one was excluded due to the absence of RV hypertrophy. Animal morphometrics are shown in Table 1 and Figure 2. Both groups injected with monocrotaline showed signs of HF-induced CC, as evidenced by RV hypertrophy, decrease in body mass and reduction in skeletal muscle (medial gastrocnemius) size (Figure 2). Additionally, we assessed exercise intolerance in rats, the primary and most debilitating symptom of HF (Table 1).

### 2.2. Differential Gene Expression Analysis Identified a Larger Transcriptome Shift in the Sedentary CC Group

To explore the molecular mechanisms contributing to exercise intolerance and skeletal muscle atrophy in our rat model of CC, we performed RNA sequencing (RNA-seq) analysis to examine the transcriptomic profile of the medial gastrocnemius muscle in both sedentary and exercised CC rats. The gastrocnemius muscle was chosen because it contains more type II (fast-twitch) muscle fibers, which are the fibers more vulnerable to chronic conditions, such as cardiac cachexia [13,14,15]. To investigate the training dependent-effects of CC on the gastrocnemius muscle, two comparisons were made: Control Sedentary × CC sedentary (Sed vs. CC-Sed) and Control Exercised × CC exercised (Ex vs. CC-Ex).

A distinct transcriptomic shift was observed in the gastrocnemius muscle of CC-Sed rats, with 131 genes upregulated and 14 genes downregulated compared to Sed group. In contrast, the transcriptomic signature in exercised CC-Ex rats compared to Ex was more modest, with only 16 genes upregulated and 2 genes downregulated (Figure 1b,c). Thirteen overlapping differentially expressed genes (DEGs) between the two comparisons were associated with gas transport (Table 2).

The top five upregulated and downregulated transcripts in the gastrocnemius muscle, based on Log2-fold change (Log2FC) and a *p*-value threshold of 0.05, for both sedentary (Sed vs. CC-Sed) and exercised (Ex vs. CC-Ex) comparisons, are shown in Table 3. The most upregulated genes in the CC-Sed group were associated with muscle structure tissue (*Tnnc1*, *Tpm3*, *Tnni1*, and *Myl3*) and cell proliferation and metabolism (*Nr4a3*). The most downregulated genes in CC-Sed are involved in cellular signaling and regulation. In contrast, the comparison between the exercised groups revealed the top four upregulated genes in CC-Ex rats to be associated with gas transport. Moreover, these top five upregulated DEGs (*Hbb*, *Hba-a1*, *Hba-a3*, *Aqp4*, and *Prodh*) in exercised groups are shared with the sedentary groups and not an exclusive signature of the CC-Ex group. The only two downregulated DEGs in CC-Ex rats were *Nrip3*, involved in proteolysis, and *Egr*1 which is related to cell growth. A full list of DEGs can be found in Supplementary Materials Table S1.

### 2.3. Pathway Enrichment Analysis of the DEGs in the Gastrocnemius Muscle of Sedentary and Exercised CC Rats

Next, a Gene Ontology (GO) [16] enrichment analysis was performed to identify biological processes (BP), cellular components, and molecular functions that were impacted in CC under sedentary or exercised conditions. Table 4 shows all cellular components and molecular functions, while Table 5 provides the top 10 BP for each comparison. Several common GO terms were identified across the Sed vs. CC-Sed and Ex vs. CC-Ex in the categories of cellular component (GO: 0005833), molecular function (GO: 0005344; 0019825), and BP (GO:0015669, GO:0015670, GO:0019755)—all of which were associated with gas transport (a full list of BP is available in the Supplementary Materials Table S2). Despite the shared terms, the overall GO enrichment profiles revealed distinct characteristics between the Sed vs. CC-Sed and Ex vs. CC-Ex comparison.

**Figure 3:**
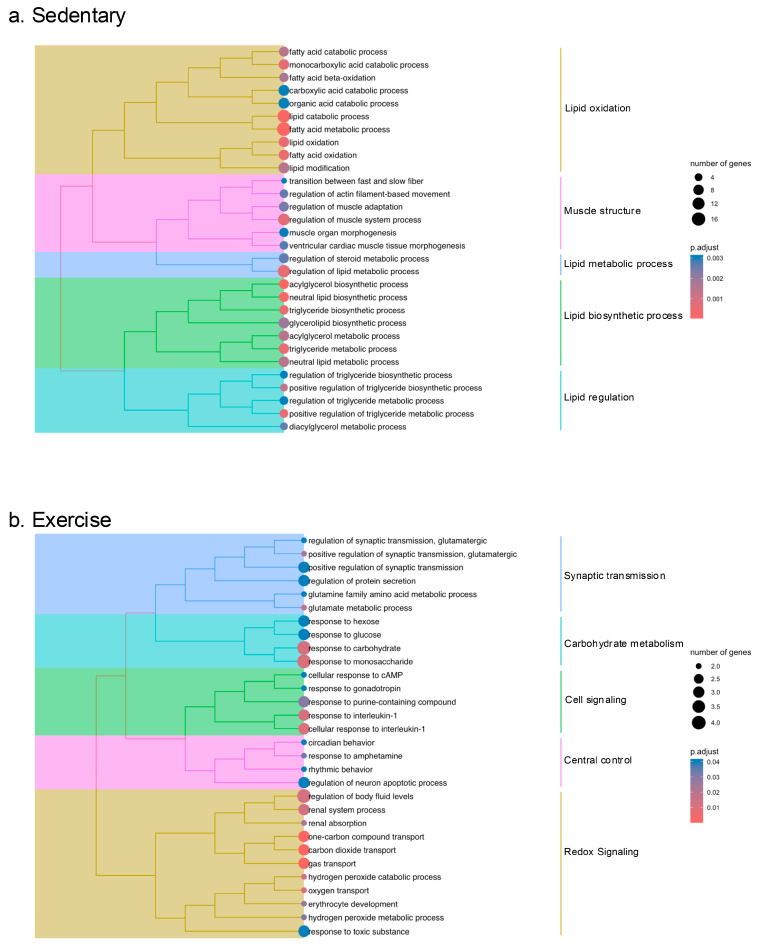
Tree plot of enriched biological process GO terms clustered by semantic similarity of (**a**) sedentary (Sed vs. CC-Sed) and (**b**) exercise (Ex vs. CC-Ex).

The tree plots in Figure 3 highlight the main themes of enriched terms of each group’s comparison. Specifically in BP, the analysis provided 114 terms significantly different in sedentary rats (Sed vs. CC-Sed), while only 37 terms were identified for the comparison between exercised rats (Ex vs. CC-Ex) (Supplementary Materials Table S2).

In the sedentary comparison, the most prominent BPs were related to lipid metabolism, including fatty acids, diacylglycerols, triglycerides, phospholipids, fat cells, adipose tissue, acyl-CoA, and carboxylic acid metabolism (Figure 3a). Key upregulated DEGs within this lipid metabolism cluster included *Dgat2* (diacylglycerol O-acyltransferase 2), *Gpam* (glycerol-3-phosphate acyltransferase, mitochondrial), *Pnpla2* (patatin-like phospholipase domain containing 2), *Slc27a1* (solute carrier family 27 member 1), and *Lpl* (lipoprotein lipase), while the only downregulated DEG was *Socs2* (suppressor of cytokine signaling 2).

**Figure 4:**
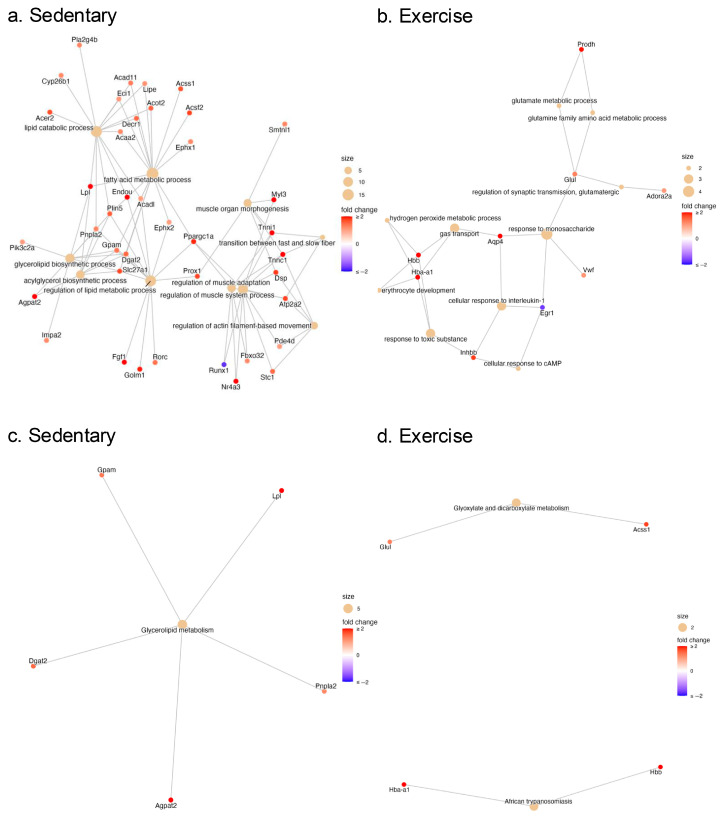
Enriched network plots: GO Biological process from (**a**) sedentary (Sed vs. CC-Sed) and (**b**) exercise (Ex vs. CC-Ex). KEGG pathway analysis from (**c**) sedentary (Sed vs. CC-Sed) and (**d**) exercise (Ex vs. CC-Ex).

The second most prominent enrichment theme in the sedentary comparison was related to muscle regulation, with BP terms involving muscle fiber types, adaptation, contraction, and actin filaments (Figure 3a). Notable upregulated DEGs in this muscle tissue theme included *Tnnc1*, *Tnni1*, and *Myl3*—which are considered the most biologically significant DEGs (see Table 3)—and *Atp2a2* (sarcoplasmic/endoplasmic reticulum Ca^2+^ ATPase). Downregulated DEGs in this theme included Runx1 and Hopx (HOP homeobox). Interestingly, *Ppargc1a* (PPARG coactivator 1 alpha) appeared in both the lipid metabolism and muscle tissue themes, as well as in other functional categories (Figure 4 and Supplementary Materials Table S2).

In contrast, the exercised comparison (Ex vs. CC-Ex) did not reveal enrichment term themes related to lipid metabolism or muscle tissue. Instead, the prominent clusters were related to synaptic transmission, carbohydrate metabolism, cell signaling, central control and redox signaling (Figure 3b). Within these clusters, the most frequently upregulated DEGs included *Hba-a1*, *Hbb*, *Hba-a3*, *Aqp4*—noted as the most biologically significant DEGs (see Table 3)—and *Glul* (glutamate-ammonia ligase), while the most frequently downregulated gene was *Egr1* (Figure 4). A detailed list of BP-related GO terms for the Ex vs. CC-Ex comparison can be found in the Supplementary Materials Table S2. 

Finally, pathway enrichment analysis was performed on the upregulated and downregulated DEGs using the Kyoto Encyclopedia of Genes and Genomes (KEGG) pathway database [17,18]. In sedentary rats, DEGs corresponded to significantly enriched terms in lipid metabolism pathways, specifically glycerolipid metabolism (rno00561). The upregulated DEGs in this pathway, including *Dgat2*, *Agpat2* (1-acylglycerol-3-phosphate O-acyltransferase 2), *Pnpla2*, *Lpl*, and *Gpam* were also found to be enriched in both GO BP and molecular functions. In contrast, the exercised rats showed enrichment in pathways related to African trypanosomiasis (rno0514), malaria diseases (rno05144), and glyoxylate and dicarboxylate metabolism (rno00630). The DEGs associated with these pathways included *Hba-a1*, *Hbb* and *Hba-a3*, and *Acss1* and *Glul* (glutamate-ammonia ligase) (Figure 3).

### 2.4. Identification of Key Regulatory Genes for Cardiac Cachexia by Weighted Gene Co-Expression Network Analysis (WGCNA)

**Figure 5:**
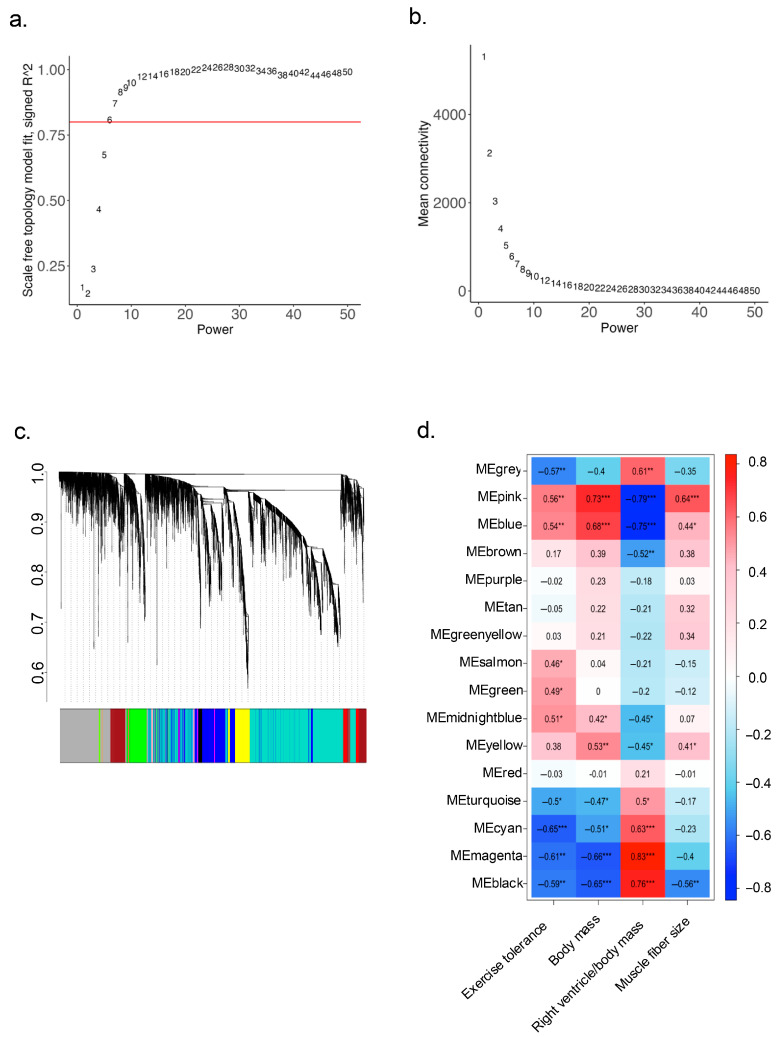
Weighted gene co-expression network analysis (WGCNA). (**a**) Scale of Independence with soft threshold power (red line); (**b**) Scale of mean connectivity; (**c**) Dendrogram highlighting color-coded module eigengenes identified by hierarchical clustering (ME); (**d**) Correlation between ME and morphometric and exercise intolerance variables. * *p* < 0.05; ** *p* < 0.001; *** *p* < 0.0001.

To identify key regulatory genes associated with CC-related outcomes observed in our animals, we performed WGNA analysis. First, we tested the distribution of samples and removed outliers. Then, we set the soft threshold for the scale of independence (Figure 5a) and mean connectivity (Figure 5b). A total of 16 modules eigengenes (ME) were identified using hierarchical clustering (Figure 5c), and the relationship between each ME and various morphometrics and exercise tolerance variables was assessed (Figure 5d).

Strong correlations were observed between several MEs and disease outcomes, particularly with body mass (MEpink r = 0.73, *p* ≤ 0.0001) and right ventricle relative mass (MEblack, r = 0.76, *p* ≤ 0.0001; MEmagenta r = 0.83, *p* ≤ 0.0001; MEblue r = −0.75, *p* ≤ 0.0001; MEpink r = −0.79, *p* ≤ 0.0001). These correlations suggest that the genes within these MEs are strongly associated with the pathophysiology of HF-induced CC. To further characterize the biological functions of the identified modules, GO enrichment analysis was performed, and up to the top 3 enriched terms for each ME are presented in Table 6.

Next, we aimed to identify potential key regulatory genes by analyzing the overlap between the DEGs in both comparison groups and the genes within the 16 identified MEs. Seven MEs were found to contain overlapping DEGs: MEpink, MEblue, MEbrown, MEyellow, MEgreenyellow, MEturquoise, and MEmagenta (Supplementary Materials Table S3). To determine the most strongly correlated overlapping DEGs, we applied a cutoff of 0.8 for the Module Membership (kME > 0.8) parameter, resulting in six remaining MEs (Table 7).

**Figure 6:**
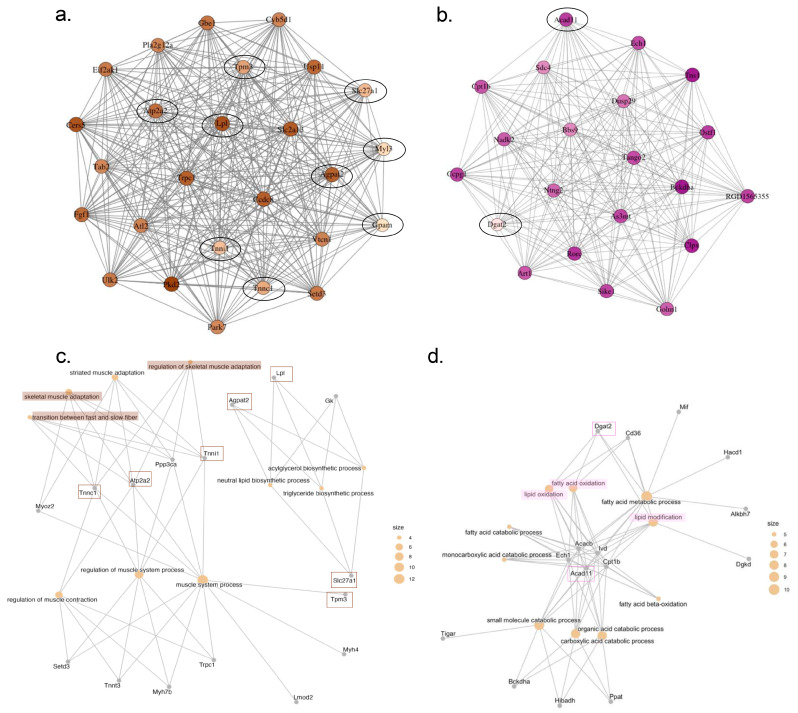
Network visualization of co-expression modules (**a**) MEbrown and (**b**) MEmagenta. Genes circled in black overlap with DEGs. Panels (**c**,**d**) show enriched GO-BPs shared between module eigengenes and DEGs for MEbrown and MEmagenta, respectively. The top three overlapping GO biological processes terms, as shown in Table 6, are highlighted. Square outlines indicate DEGs connected to the top three overlapping GO biological processes.

Additionally, MEbrown and MEmagenta presented some BPs that were also found in DEGs, as shown in Figure 6. Interestingly, the BPs of MEbrown are mostly involved with muscle tissue and secondly involving lipid metabolism, with the overlapping DEGs *Tnnc1*, *Tnni1*, *Tpm3*, *Atp2a2*, *Lpl*, *Agpat2, Slc27a1*, while in MEmagenta the BPs broadly involve substrates metabolism with mostly lipid metabolism, which *Acad11* and *Dgat2* are the more frequent overlapping DEGs.

Integrating the findings from the differential gene expression, GO, KEGG, and WGCNA analyses, we identified several hub genes crucial to the pathophysiology of HF-induced CC. These include: *Dgat2*, *Lpl*, *Pnpla2*, Agpat2, *Slc27a1*, *Tnnc1*, *Tnni1*, *Myl3*, *Tpm3*, *Atp2a2*, *Vwf* and *Prodh*. Additionally, we highlight *Fbxo32* (F-box Protein 32) and *Acad11* (acyl-CoA dehydrogenase family member 11), as they are involved in important pathways related to protein degradation and fatty acid oxidation, respectively.

### 2.5. Validation of DEGs Related to Lipid Metabolism

**Figure 7:**
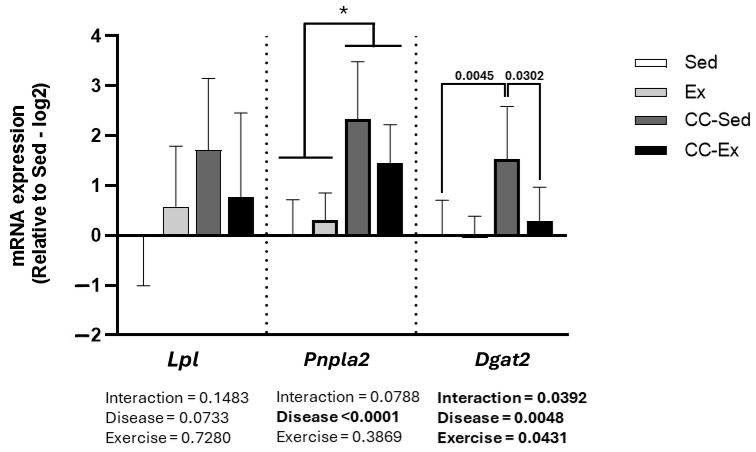
Validation of DEGs related to lipid metabolism in medial gastrocnemius muscle. Sed: control sedentary group; Ex: control exercised; CC-Sed cardiac cachexia sedentary group; CC-Ex: cardiac cachexia exercised group. * Main effect of Disease.

As shown in Table 6, all overlapping DEGs—except for *Vwf* and *Prodh*—originated from the sedentary comparison group. This finding supports the hypothesis that aerobic training mitigates the negative transcriptional effects of HF-induced CC, particularly those impacting lipid metabolism. To test this hypothesis, we performed real-time quantitative polymerase chain reaction (RT-qPCR) on three DEGs related to lipid metabolism: *Lpl* (lipid uptake), *Dgta2* (lipogenesis), and *Pnpla2* (lipolysis) in the skeletal muscle. As shown in Figure 7, these genes showed differential expression trends. Significant disease effects (Control vs. CC) were observed for *Pnpla2* (F _(1,23)_ = 29.39, *p* < 0.0001) and *Dgta2* (F _(1,23)_ = 9.719, *p* = 0.0048), with a trend toward significance for *Lpl* (F _(1,23)_ = 3.507, *p* = 0.0733), indicating that CC markedly affected lipid metabolism. Importantly, *Dgta2* was the only gene to show a significant interaction effect, with significant difference between CC-Sed and CC-Ex groups (F _(1,23)_ = 4.779, *p =* 0.0302). This finding suggests that aerobic training normalized the gene expression of *Dgat2*, a key enzyme in lipogenesis, in the skeletal muscle of rats with CC.

## 3. Discussion

This study presents the first comparative RNA-seq analysis of the medial gastrocnemius muscle in sedentary and exercised rats with HF-induced CC. Our primary objective was to investigate the therapeutic effects of aerobic training on transcriptomic alterations associated with disease progression in cachectic skeletal muscle. We identified distinct transcriptomic profiles between sedentary and exercised rats. Functional and pathway enrichment analyses revealed genes involved in fatty acid metabolism and muscle tissue remodeling in sedentary cachectic muscles—an effect not observed in the exercised ones. Subsequently, weighted gene co-expression network analysis (WGCNA) demonstrated strong correlations between transcriptomic modules and phenotypic traits such as body mass, relative right ventricular mass, exercise tolerance, and muscle fiber size cross-sectional area. Finally, since lipid metabolism seemed to have been more affected by HF-induced CC, we validated three hub genes engaged with lipid metabolic process in skeletal muscle: uptake (*Lpl*), lipogenesis (*Dgat2*) and lipolysis (*Pnpla2*). Our findings provide molecular insights into the protective role of aerobic training against the muscle alterations induced by CC and may inform the development of targeted therapeutic approaches for preserving skeletal muscle function in chronic HF.

We used monocrotaline-induced pulmonary arterial hypertension as the experimental model of HF-induced CC, as it rapidly induces progressive heart failure and cachexia [19]. Pulmonary arterial hypertension affects the right side of the heart, causing pathological hypertrophy and dysfunction of the RV due to pressure overload [20]. While both ventricles are influenced by loading conditions, the RV is especially sensitive to [21] afterload; even small increases can significantly impair its function. Moreover, right-sided heart failure often results from left-sided heart failure, also known as congestive heart failure [22]. Although left ventricle failure may show a more pronounced skeletal muscle impairment compared to RV failure, both left- and right-sided HF can lead to skeletal muscle wasting and dysfunction, and ultimately cardiac cachexia [1,5,21]. In this study, we examined the therapeutic effects of an aerobic program, initiated alongside disease progression, on HF outcomes and skeletal muscle transcriptomic alterations in our preclinical model of right-sided HF-induced CC.

Aerobic training was implemented over a 4-week period, coinciding with the development of CC. Animal with HF-induced CC exhibited characteristic signs, such as body mass loss, increased right ventricular mass, skeletal muscle atrophy, and reduced exercise tolerance (Table 1). Aerobic training did not significantly change the clinical indicators of CC, which may be due to the short duration of the four-week training particularly in a rapidly progressing model like the monocrotaline-induced HF. However, molecular analysis of skeletal muscle differed substantially between sedentary and exercised animals. Our RNA-seq analysis identified 145 differentially expressed genes (DEGs) between sedentary groups, compared to only 18 DEGs between exercise groups. This suggests that aerobic training during the disease progression mitigated numerous transcriptomic alterations characteristic of sedentary cachectic muscles, indicating that molecular adaptations may precede detectable physiological improvements. Our findings reinforce the scientific evidence supporting the role of aerobic training in the treatment and management of patients with HF induced by pulmonary arterial hypertension, as recommended by clinical guidelines [7,8], by providing objective evidence that its benefits extend to the transcriptional level. To investigate this further, a time-course study examining the evolving transcriptional landscape in response to exercise during disease progression would be highly beneficial. In addition, we hypothesize that an aerobic training protocol lasting more than four weeks may allow transcriptomic alterations to manifest as physiological and phenotypic improvements. Therefore, further studies in this area of research are highly promising.

Exercise intolerance is the primary clinical manifestation of CC and results, at least in part, from structural, functional, and metabolic changes that occur in skeletal muscle during HF [6]. Specifically, metabolic alterations impair muscle contraction and relaxation, promote muscle fiber type transition, and contribute to muscle atrophy [23]. It is well established that, in pathological conditions, such as HF, slow-twitch fibers tend to shift toward fast-twitch fibers, which are more susceptible to atrophy [6,15,23,24]. One of the key drivers of skeletal muscle atrophy is the E3 ligase enzyme atrogin-1, encoded by the *Fbxo32* gene, which is part of the ubiquitin-proteasome system. Consistent with previous studies, our results show upregulation of *Fbox32* in sedentary cachectic muscles only [12,15,25,26]. Our data further revealed that *Fbxo32* may serve as a potential hub gene, present in the MEturquoise, and correlated with body mass, relative right ventricle mass and exercise intolerance (Figure 4). Notably, *Fbxo32* was not differentially expressed between the exercise groups, supporting the potential capacity of aerobic training to mitigate ubiquitin-proteasome activity [27] by normalizing expression of this CC-associated hub gene.

Surprisingly, the most biologically significant DEGs in sedentary groups (Table 3) were genes encoding proteins associated with slow-twitch muscle fibers, such as *Tnnc1*, *Tnni1*, *Myl3*, *Tpm3* and *Atp2a2*. While this might suggest increased turnover of slow-twitch fibers, previous studies [12,25,28] indicate the opposite—an upregulation of genes and proteins related specifically to fast-twitch fiber type in disease conditions. This suggests that these slow-twitch-associated genes may instead be involved in signaling pathways unrelated to type I fiber remodeling. Troponin and tropomyosin isoforms, for instance, have been implicated in nuclear functions, transcription factors, atrophy, and muscle weakness [29,30,31], which may explain the elevated expression of *Tnnc1*, *Tnni1* and *Tpm3* in sedentary cachectic muscles observed in our study. In contrast, their lack of upregulation in the cachectic rats of the exercise group may be attributed to the effects of aerobic training. Cheung et al. [31], using an IL-1 receptor agonist, demonstrated that anti-inflammatory therapy normalized *Tnnc1*, *Tpm3* and *Atp2a2* expression in the gastrocnemius muscle of mice with chronic kidney disease, suggesting a potential therapeutic approach for muscle wasting. This supports the idea that aerobic training—known for its anti-inflammatory effects [32]—may prevent the upregulation of these genes in exercised cachectic rats.

Furthermore, we identified an upregulation of several genes associated with lipid metabolism through functional pathway (GO and KEGG) enrichment analyses. These results suggest possible dysfunctions in lipid storage, oxidation, and other metabolic processes. This was further supported by WGCNA, which identified key hub genes involved in lipid metabolism, including *Lpl*, *Pnpla2*, *Agpat2*, *Slc27a1*, *Acad11* and *Dgat2*. The GO biological processes (BPs) from the WGCNA also showed significant enrichment in pathways related to lipid metabolism (Figure 5). These results could be in accordance with evidence described in the current literature. Preclinical and clinical studies have demonstrated that the infiltration and accumulation of ectopic fat—particularly in skeletal muscle, a condition known as myosteatosis—can impair muscle metabolism and function under pathophysiological conditions, such as heart disease [33,34].

When analyzed in detail, the hub genes related to lipid metabolism identified in this study seem to follow a sequence of events which point to the fat accumulation and oxidation in skeletal muscle, as described as follows. *Lpl* encodes the protein lipoprotein lipase, which is an enzyme that facilitates the hydrolysis of the extracellular triacylglycerol (triglycerides) into three fatty acids and one glycerol molecule. *Slc27a1* encodes the fatty acid transporter member 1, a key membrane transporter, that enables fatty acids to enter the cytosol. *Agpta2* and *Dgat2* encode enzymes that convert cytosolic fatty acids and glycerol-3-phosphate into intracellular triacylglycerol, which is stored as lipid droplets within muscle fibers [35]. Lipid droplet formation is known to function as a protective mechanism against lipotoxicity, particularly under pathological conditions of cellular stress or excess fatty acids [36]. Therefore, we hypothesized that the observed upregulation of *Lpl* and *Slc27a1* could represent a response to dyslipidemia triggered by circulating pro-inflammatory molecules in CC [37,38], while *Agpat2* and *Dgat2* may reflect a muscle adaptive response aimed at promoting lipid storage within the skeletal muscle to buffer against lipotoxicity-induced muscle damage in sedentary cachectic muscles. A concurrent upregulation of *Plin 5* (perilipin 5)—a protein associated with lipid droplet stabilization and protection—seen in the sedentary CC group [35] further could support this interpretation (Table 5 and Figure 3).

While lipid droplet formation may offer protective benefits, excessive lipid accumulation (e.g., myosteatosis) can have detrimental effects. When lipid storage becomes dysregulated, it may lead to inappropriate activation of lipogenesis, lipolysis and lipid oxidation pathways. This dysregulation can result in the accumulation of lipotoxic intermediates, such as diacylglycerol and ceramides, which are associated with oxidative stress and pro-inflammatory signaling—processes implicated in muscle atrophy, particularly in the context of cachexia [39]. Our study showed evidence supporting this possibility in transcriptional level, which include the upregulation of *Pnpla2*, a gene that encodes the triacylglycerol lipase, an intracellular enzyme responsible for hydrolyzing triacylglycerol into diacylglycerol, a potentially harmful intermediate if accumulated in excess [35]. In addition, we also found an upregulation of *Acad11*, a gene that encodes the acylCoA dehydrogenase family member 1, a mitochondrial enzyme [40]. These DEGs might indicate an attempt to improve lipid oxidation activity, which can be contributing to skeletal muscle dysfunction in sedentary CC rats. Notably, these DEGs involved with lipid metabolism were not upregulated in exercised cachectic rats. Given the metabolic demand of aerobic exercise, which relies heavily on lipid oxidation, it is plausible that physical activity could help normalize lipid metabolism and reduce the risk of lipotoxicity-induced muscle damage.

Thus, we performed the validation of three of these DEGs (*Lpl, Dgat2*, *Pnpla2*) to investigate further the lipid metabolism in skeletal muscle in HF-induced CC. The PCR analysis showed in fact there is an altered lipid metabolic process in HF-induced CC groups when compared with the control groups and *Dgat2* was normalized by aerobic training. Although this validation points out to the therapeutic effects of this exercise intervention, further analyses including protein expression and histological protocols are needed to confirm the hypothesis. In view of the HF-induced CC model utilized which allowed four weeks of exercise intervention, we believe that long-term aerobic training may normalize all hub genes found in this study. In addition, other varieties of volume and intensity should be considered in this same model. Thus, future research focusing on biochemical and histological analyses is needed to uncover if the transcriptional outcomes are being translated phenotypically, and then to support this hypothesis.

In the present study, we identified 13 DEGs common to both sedentary and exercise comparisons, suggesting that the 4-week aerobic training program did not normalize their expression (Table 2). Several of these DEGs were involved in gas transport and were among the most biologically significant in the exercised cachectic group (Table 2 and Table 3). WGCNA identified *Vwf* and *Prodh* as hub genes in the MEpink module, which correlated to body mass, right ventricular mass, muscle fiber size, and reduced exercise tolerance. *Vwf*, a key factor in hemostasis, is upregulated in response to hypoxia and inflammation [41], aligning with known vascular alterations and systemic inflammation in HF [6,41]. This may explain its persistent elevation in both sedentary and exercised CC groups. *Prodh*, a mitochondrial enzyme involved in energy metabolism, redox balance, and apoptosis regulation, is typically downregulated in dysfunctional disease states, while exercise seems to increase its expression [42,43,44]. Interestingly, we observed *Prodh* upregulation in skeletal muscle in both sedentary and exercised CC groups. Given the limited literature on *Prodh* and *Vwf* in skeletal muscle under cachectic conditions, further studies are needed to clarify their roles in disease progression and exercise response.

An important limitation in this study was the absence of improvements in body mass, relative right ventricular mass, muscle fiber size, and exercise tolerance in the exercised cachectic group. Although such functional enhancements are typically expected with aerobic training, our 4-week training protocol may not have been the appropriate endpoint to elicit measurable phenotypic changes in this rapidly progressing model of HF-induced cachexia. Models featuring more gradual disease progression, such as some types of genetic and surgical models (see Molinari et al. [19]), may offer a better timeframe for detecting physiological improvements. In addition, protocols involving an earlier exercise training intervention (before and during the development of HF) can be an alternative to assess the long-term effects aerobic training on the monocrotaline model of HF-induced CC. Moreover, impairments in downstream processes such as protein translation, synthesis, and post-translational modifications [45] could be hindering the manifestation of functional improvements, despite transcriptional normalization. Another important consideration is that validation by RT-PCR was applied not for all the hub genes suggested in the present study; however, the bioinformatic tool WGCNA provided strong evidence for the involvement of these key regulatory genes. Finally, although muscle remodeling and lipid metabolism are discussed, the present study did not include histological or functional assessments of skeletal muscle, such as fiber type composition, intramuscular fat quantification, or contractile performance, which limits the ability to directly correlate transcriptomic findings with morphological or physiological outcomes.

In conclusion, this study demonstrated, through RNA-seq analysis and bioinformatic tools, that aerobic training prevented alterations in the expression of genes associated with skeletal muscle dysfunction in CC, particularly those involved in muscle remodeling and mainly lipid metabolism. Validation of *Dgat2* supports a protective transcriptional effect of aerobic training. These findings provide a foundation for future research to further explore the pathways and mechanisms highlighted by our RNA-seq data, validate these results at the protein level, and better understand translational mechanisms. Moreover, exploring alternative exercise protocols and durations may further optimize exercise-based interventions for CC.

## 4. Materials and Methods

All animal procedures were approved by the Institutional Animal Care and Use Committee of Texas A&M University, TX, USA (IACUC 2023-0035), which follows the Animal Welfare Act.

### 4.1. Animals

40 male Wistar rats weighing 165–175 g were acquired from Charles River Laboratories (Wilmington, MA, USA). Upon arrival, rats were housed two per cage in ventilated racks into a reverse dark-light cycle housing with lights off at 7:00 AM and lights on at 7:00 PM. The animal housing room was monitored daily and maintained at 22 ± 1 °C and ~60% humidity. Red lights were used during the dark cycle to provide adequate lighting for staff to perform rodent handling and exercise training; no standard lighting was used during the dark phase. After one week of acclimation period, rats were randomly assigned into four groups based in their body mass: control sedentary (Sed, *n* = 9), control exercised (Ex, *n* = 9), cardiac cachexia sedentary (CC-Sed, *n* = 12) and cardiac cachexia exercised (CC-Ex, *n* = 10). Rats in CC-Sed and CC-Ex groups received a single injection of monocrotaline (I.P., 60 mg/kg, MedChemExpress, Monmouth Junction, NJ, USA) for the induction of HF, while rats in both control groups (Sed and Ex) received an equivalent volume of saline as a vehicle control (Figure 1a). At the end of the experiment, the criteria applied to determine the presence of CC included exercise intolerance, apathy, lethargy, and morphological parameters including >8.5% of weight loss (from the peak of body mass), right ventricle (RV) hypertrophy, increased total heart mass, and organ congestion.

### 4.2. Treadmill Familiarization, Exercise Tolerance Capacity Test, and Aerobic Training

After three consecutive days of treadmill familiarization (5 min at 5 m/min), all animals underwent a maximal incremental exercise test pre- and post-training to evaluate their exercise tolerance (time to fatigue) and to determine the training speed for exercise groups (Ex and CC-Ex). The maximal incremental exercise test consisted of 5 min warm-up at 5 m/min, followed by increments of 3 m/min every three minutes until rats presented signs of exhaustion (refusing to run even with manual and electric stimulation, or being unable to coordinate steps). Maximum speed, total time and distance were recorded. Aerobic training was performed under red lights during the dark cycle, 5 consecutive days per week followed by 2 rest days. Training protocol was performed on a treadmill and consisted of 5 min warm-up at 5 m/min, 30 min at 50–60% of maximal capacity, and 5 min cool down, 5×/week for four weeks (Figure 1a), when the rats which received monocrotaline presented a significant decline of health conditions (apathy, piloerection, tachypnea and exercise intolerance).

### 4.3. Euthanasia and Tissue Collection

Euthanasia was performed at least 48 h after the final maximal exercise capacity test with an intraperitoneal injection of pentobarbital sodium (50 mg/kg) (Figure 1a). Cardiac exsanguination was used as the secondary method to confirm euthanasia. After blood collection, the heart was removed by thoracotomy. Ventricles were dissected and weighed; right ventricle weight was used to estimate cardiac hypertrophy by the RV/body mass ratio (mg/g). Gastrocnemius muscles (medial head) from the right and left hind limbs were dissected, weighed, and frozen in cold isopentane for histological analysis or snap frozen in cold liquid nitrogen, and stored at −80 °C for further transcriptome analysis, respectively.

### 4.4. Skeletal Muscle Cross-Sectional Area

To evaluate muscle fiber size, 10 μm-thick cross-sections were cut from the mi-belly of the medial gastrocnemius muscle using a cryostat (Shandon Inc., Pittsburgh, PA, USA) cooled at −20 °C. Briefly, sections were incubated in anti-laminin (1:500; Invitrogen, Catalog # PA1-16736) for 1 h at room temperature (RT). Sections were washed and incubated in the secondary antibody (goat anti-rabbit Alexa Fluor 405; 1:250; Invitrogen, Waltham, MA, USA, Catalog # A-31556) for 45 min at RT. Slides were mounted with ProLong^TM^ Gold Antifade Mountant (Invitrogen, Catalog # P36934) and stored at 4 °C until imaging. Sections were imaged using the Leica DMi8 confocal microscope (Leica Microsystems, Deerfield, IL, USA). Image J 1.54f software (Java 1.8.0_322 64-bit, NIH, Bethesda, MD, USA) was used to measure and calculate the average area of approximately 200 fibers from each animal.

### 4.5. RNA Isolation and RNA-Seq Library Preparation

Total RNA was extracted from 30 mg of frozen medial gastrocnemius (left leg) of six samples per group using the RNeasy^®^ Plus Universal mini kit (QIAGEN, Hilden, Germany) following the manufacturer’s instruction. Total RNA quality was assessed using the Agilent TapeStation (Santa Clara, CA, USA), and all samples had RNA integrity numbers (RIN) greater than 8. RNA quantification was performed with the Qubit RNA BR Assay Kit. Poly-A-based RNA-seq libraries were prepared using the Illumina Stranded mRNA Prep Kit (San Diego, CA, USA) with 650 ng of RNA input per sample, following the manufacture’s protocol. Library quality and size distribution (average size: 340–360 bp) were confirmed using the Agilent TapeStation. Sequencing was performed on an Illumina NextSeq 2000 platform configured for paired-end 2 × 100 reads.

### 4.6. RNA-Seq Data Processing and Differential Expression Analysis

Reads were trimmed of low-quality (Phred < 20), adapter, and barcode sequences using Trim-Galore [46]. Sequences were aligned to the Rattus norvegicus transcriptome (Ensembl mRatBN7.2.112) and quantified at the transcript level using Salmon V1.10.2 [47] (Rob Patro 2017). Differential expression was conducted using the R 4.3.3 programming language. Briefly, transcript-level abundances generated by Salmon were collapsed down to the gene level using the “tximport 1.36.0” package from BioconductoR 3.21, by a transcript-to-gene mapping built using the R. norvegicus (Ensembl mRatBN7.2.112) GFF3 annotation file with the “txdbmaker 1.20.0” BioconductoR package.

Statistical testing was conducted with DESeq2 (v1.44.0) [48]. Significant genes were defined as those with an adjusted *p*-value < 0.05 (BH corrected, Wilcoxon p-test) and a log2 fold-change magnitude of 1 or greater. Pathway enrichment was carried out using clusterProfiler (v4.12.6) [49] with both Kyoto Encyclopedia of Gene and Genomes (KEGG) [17,18] and Gene Ontology (GO) [16] enrichment analysis for R. norvegicus pathway databases with a *p*-adjusted value < 0.05 (BH corrected).

### 4.7. WGCNA

Co-expression networks were constructed using WGCNA (v 1.73) package in R. Low-expression genes were filtered, retaining those with ≥15 counts in samples. A signed network was constructed using a soft-thresholding power of 12, selected based on scale-free topology criteria. Modules were identified using the blockwiseModules function with TOMType = “signed” and mergeCutHeight = 0.25. Module eigengenes were correlated with sample traits to identify biologically relevant modules. Hub genes within each module were ranked based on module membership (kME) and intramodular connectivity (kWithin) [50].

### 4.8. Real Time Quantitative Polymerase Chain Reaction (RT-qPCR)

Total RNA was extracted and quantified as described above in Section 4.5. Approximately 1 μg of RNA was reverse transcribed using High-Capacity cDNA Reverse Transcription Kit (Applied Biosystem, Foster City, CA, USA), according to manufacturer’s instructions. RT-PCR was carried out in 20 μL reactions using TaqMan™ Fast Advanced Master Mix for qPCR and TaqMan™ gene expression assays for *Lpl* (Rn00561482_m1, Applied Biosystem), *Dgat2* (Rn01506787_m1, Applied Biosystem) and *Pnpla2* (Rn01479969_m1, Applied Biosystem). The amplification and analysis were performed using QuantStudio^TM^ 6 Pro Real-Time PCR System (Applied Biosystem), following the manufacturer’s recommendations. The CT comparative method (2^−∆∆Ct^) was used to determine gene expression changes between treatment groups relative to the control sedentary (Sed) group, with the GAPDH Ct (Rn01775763_g1, Applied Biosystem) as the correction factor, using the software Design & Analysis 2 (Thermo Fisher Scientific, Waltham, MA, USA).

### 4.9. Statistical Analysis

Comparisons between the four groups were performed by two-way analysis of variance followed by Tukey’s post hoc test. Data normality was evaluated by Shapiro–Wilk test. All variables (body mass, RV/body mass, exercise tolerance, cross-sectional area, log2 *Lpl*, log2 *Dgat2* and log2 *Pnpla2*) were classified as parametric variables. The significance levels were set at 5%.

## Figures and Tables

**Table 1:** Indicators of Heart Failure and Cardiac Cachexia.

	Sed *n* = 9	Ex *n* = 8	CC-Sed *n* = 7	CC-Ex *n* = 6		*p*-Value
Body mass (g)	410.3 ± 29.5	392.6 ± 26.1	331.5 ± 31.3	338.7 ± 39.8	Interaction	*p* = 0.2918
					Exercise	*p* = 0.6538
					Disease	***p < 0.0001***
Relative RV mass (mg/g)	0.514 ± 0.07	0.491 ± 0.03	1.175 ± 0.20	1.188 ± 0.20	Interaction	*p* = 0.7402
					Exercise	*p* = 0.9233
					Disease	***p < 0.0001***
Exercise Tolerance (min)	16.97 ± 3.4	23.54 ± 2.9 *	11.40 ± 2.6 *^#^	10.26 ± 3.8 *^#^	Interaction	***p = 0.0036***
					Exercise	***p = 0.0320***
					Disease	***p < 0.0001***

Values presented as mean ± standard deviation. Sed: control sedentary group; Ex: control exercised; CC-Sed cardiac cachexia sedentary group; CC-Ex: cardiac cachexia exercised group; RV: Right ventricle. Muscle fiber size refers to medial gastrocnemius muscle cross-sectional area. Exercise tolerance refers to running time to fatigue. Relative RV mass: total RV mass/total body mass (mg/g) * *p* < 0.05 vs. S group; ^#^ *p* < 0.05 vs. Ex group. Significant *p*-Value are highlighted.

**Table 2:** Shared differentially expressed genes (DEGs) between sedentary and exercise comparison.

	Sedentary		Exercise		Description
Symbol	Log2 FC	*p*-Adjusted	Log2 FC	*p*-Adjusted	
*Acer2*	1.66966	4.30 × 10^−9^	1.66688	3.14 × 10^−9^	alkaline ceramidase 2
*Acss1*	1.63399	0.00060	1.78190	0.00023	acyl-CoA synthetase short-chain family member 1
*Adora2a*	1.04996	0.00374	1.04944	0.01295	adenosine A2a receptor
*Aqp4*	1.99764	0.02221	2.03651	0.04934	aquaporin 4
*Hba-a1*	1.71166	0.00391	2.45445	1.08 × 10^−5^	hemoglobin alpha, adult chain 1
*Hbb*	1.76343	0.00642	2.62157	1.13 × 10^−5^	hemoglobin subunit beta
*Inhbb*	1.62991	0.00091	1.79615	0.00034	inhibin subunit beta B
*Ppp1r3c*	1.25889	0.00389	1.18808	0.01937	protein phosphatase 1, regulatory subunit 3C
*Prodh*	2.36806	0.00013	1.96116	0.00442	proline dehydrogenase
*Tmem252*	1.44794	0.00091	1.12137	0.03606	transmembrane protein 252
*Tp53i11*	1.18541	0.01490	1.43972	0.00680	tumor protein p53 inducible protein 11
*Vwf*	1.35910	0.00091	1.10128	0.02476	von Willebrand factor

Sedentary = Sed vs. CC-Sed. Exercise = Ex vs. CC-Ex.

**Table 3:** The most biologically significant DEGs in sedentary and exercised comparison.

		Symbol	Log2FC	Name
Sedentary	Upregulated	*Tnnc1*	4.06665	troponin C1, slow skeletal and cardiac type
		*Nr4a3*	4.00686	nuclear receptor subfamily 4, group A, member 3
		*Tpm3*	3.94351	tropomyosin 3
		*Tnni1*	3.89482	troponin T1, slow skeletal type
		*Myl3*	3.73428	myosin light chain 3
	Downregulated	*Runx1*	−1.74698	RUNX family transcription factor 1
		*Bhlhe41*	−1.37910	basic helix-loop-helix family, member e41
		*Slc25a30*	−1.36981	solute carrier family 25, member 30
		*Tnfrsf21*	−1.36134	TNF receptor superfamily member 21
		*Map3k14*	−1.34111	mitogen-activated protein kinase kinase kinase 14
Exercise	Upregulated	*Hbb*	2.62157	hemoglobin subunit beta
		*Hba-a1*	2.45445	hemoglobin alpha, adult chain 1
		*Hba-a3*	2.42841	hemoglobin alpha, adult chain 3
		*Aqp4*	2.03651	aquaporin 4
		*Prodh*	1.96115	proline dehydrogenase
	Downregulated	*Nrip3*	−2.62039	nuclear receptor interacting protein 3
		Egr1	−1.68517	early growth response 1

Sedentary = Sed vs. CC-Sed. Exercise = Ex vs. CC-Ex.

**Table 4:** List of significantly enriched GO terms in sedentary and exercise comparisons.

Sedentary				Exercise			
GO ID	Cellular Component	*p*-Adj	GeneID	GO ID	Cellular Component	*p*-Adj	GeneID
0005833	Hemoglobin complex	0.025	*Hba-a1/Hbb/Hba-a3*	0005833	Hemoglobin complex	0.000	*Hba-a1/Hbb/Hba-a3*
				0097386	Glial cell projection	0.000	*Aqp4/Glul*
**GO ID**	**Molecular Function**	***p*-Adj**	**GeneID**	**GO ID**	**Molecular Function**	***p*-Adj**	**GeneID**
0015645	fatty acid ligase activity	0.007	*Acsf2/Acss3/Acss1/Slc27a1*	0005344	oxygen carrier activity	0.000	*Hba-a1/Hbb/Hba-a3*
0016405	CoA-ligase activity	0.007	*Acsf2/Acss3/Acss1/Slc27a1*	0019825	oxygen binding	0.000	*Hba-a1/Hbb/Hba-a3*
0016878	acid-thiol ligase activity	0.007	*Acsf2/Acss3/Acss1/Slc27a1*	0004601	peroxidase activity	0.000	*Hba-a1/Hbb/Hba-a3*
0016877	ligase activity, forming carbon-sulfur bonds	0.019	*Acsf2/Acss3/Acss1/Slc27a1*	0016684	oxidoreductase activity, acting on peroxide as acceptor	0.000	*Hba-a1/Hbb/Hba-a3*
0004806	triglyceride lipase activity	0.029	*Lpl/Lipe/Pnpla2*	0016209	antioxidant activity	0.001	*Hba-a1/Hbb/Hba-a3*
0016411	acylglycerol O-acyltransferase activity	0.030	*Dgat2/Agpat2/Pnpla2*	0140104	molecular carrier activity	0.001	*Hba-a1/Hbb/Hba-a3*
0009975	cyclase activity	0.030	*Dglucy/Gucy2g/Npr2*	0020037	heme binding	0.006	*Hba-a1/Hbb/Hba-a3*
0008374	O-acyltransferase activity	0.030	*Gpam/Dgat2/Agpat2/Pnpla2*	0046906	tetrapyrrole binding	0.006	*Hba-a1/Hbb/Hba-a3*
0019825	oxygen binding	0.033	*Hba-a1/Hbb/Hba-a3*	0043177	organic acid binding	0.000	*Prodh/Hba-a1/Hbb/Hba-a3/Glul*
0005344	oxygen carrier activity	0.019	*Hba-a1/Hbb/Hba-a3*	0001664	G protein-coupled receptor binding	0.020	*Adora2a/Hba-a1/Hba-a3*
				0016597	amino acid binding	0.015	*Prodh/Glul*

GO: Gene Ontology. Sedentary = Sed vs. CC-Sed. Exercise = Ex vs. CC-Ex.

**Table 5:** Top 10 GO biological process enriched terms.

Sedentary Groups Comparison (S vs. CC-S)			
GO ID	Biological Process (BP)	*p*-Adj	GeneID
GO:0006631	fatty acid metabolic process	0.000	*Acsf2/Ephx1/Ppargc1a/Nr4a3/Acss1/Decr1/Eci1/Acot2/Acad11/Lpl/Acadl/* *Acaa2/Gpam/Dgat2/Ephx2/Slc27a1/Lipe/Plin5*
GO:0044242	cellular lipid catabolic process	0.000	*Pla2g4b/Acer2/Decr1/Eci1/Acad11/Lpl/Acadl/Acaa2/Cyp26b1/Lipe/Plin5/Pnpla2*
GO:0019395	fatty acid oxidation	0.000	*Ppargc1a/Nr4a3/Decr1/Eci1/Acad11/Acadl/Acaa2/Dgat2/Plin5*
GO:0016042	lipid catabolic process	0.000	*Pla2g4b/Acer2/Decr1/Eci1/Acot2/Acad11/Lpl/Acadl/Acaa2/Cyp26b1/Lipe/Plin5/Endou/Pnpla2*
GO:0046460	neutral lipid biosynthetic process	0.000	*Lpl/Gpam/Dgat2/Slc27a1/Agpat2/Plin5/Pnpla2*
GO:0046463	acylglycerol biosynthetic process	0.000	*Lpl/Gpam/Dgat2/Slc27a1/Agpat2/Plin5/Pnpla2*
GO:0034440	lipid oxidation	0.000	*Ppargc1a/Nr4a3/Decr1/Eci1/Acad11/Acadl/Acaa2/Dgat2/Plin5*
GO:0019216	regulation of lipid metabolic process	0.000	*Prox1/Ppargc1a/Nr4a3/Acadl/Fgf1/Gpam/Dgat2/Ephx2/Slc27a1/Golm1/Rorc/Plin5/Endou/Pnpla2*
GO:0019432	triglyceride biosynthetic process	0.000	*Lpl/Gpam/Dgat2/Slc27a1/Agpat2/Plin5*
GO:0090208	positive regulation of triglyceride metabolic process	0.001	*Gpam/Dgat2/Slc27a1/Plin5/Pnpla2*
**Exercise Groups Comparison** **(Ex vs. CC-Ex)**			
**GO ID**	**Biological Process (BP)**	***p*** **-Adj**	**GeneID**
GO:0015669	gas transport	0.000	*Aqp4/Hba-a1/Hbb/Hba-a3*
GO:0015670	carbon dioxide transport	0.000	*Aqp4/Hba-a1/Hbb*
GO:0015671	oxygen transport	0.000	*Hba-a1/Hbb/Hba-a3*
GO:0019755	one-carbon compound transport	0.000	*Aqp4/Hba-a1/Hbb*
GO:0042744	hydrogen peroxide catabolic process	0.000	*Hba-a1/Hbb/Hba-a3*
GO:0042743	hydrogen peroxide metabolic process	0.001	*Hba-a1/Hbb/Hba-a3*
GO:0098869	cellular oxidant detoxification	0.005	*Hba-a1/Hbb/Hba-a3*
GO:0009636	response to toxic substance	0.008	*Hba-a1/Hbb/Hba-a3/Inhbb*
GO:1990748	cellular detoxification	0.008	*Hba-a1/Hbb/Hba-a3*
GO:0034284	response to monosaccharide	0.009	*Aqp4/Egr1/Vwf/Glul*

GO: Gene Ontology. Sedentary = S vs. CC-S. Exercise = Ex vs. CC-Ex.

**Table 6:** Top three GO biological process enriched terms for ME.

ME	Biological Process	*p*-Adj
MEgrey	regulation of mitochondrial membrane potential	0.000788
	glial cell activation	0.010507
	negative regulation of amyloid fibril formation	0.014577
MEpink	mitochondrial membrane organization	0.039761
	inner mitochondrial membrane organization	0.043771
MEblue	purine ribonucleotide catabolic process	0.008092
	ribonucleotide catabolic process	0.008092
	purine nucleotide catabolic process	0.008092
MEbrown	transition between fast and slow fiber	0.000257
	skeletal muscle adaptation	0.000402
	regulation of skeletal muscle adaptation	0.000402
MEpurple	cell proliferation in external granule layer	0.007065
	cerebellar granule cell precursor proliferation	0.007065
	cell proliferation in hindbrain	0.007065
MEgreenyellow	microautophagy	0.013788
	protein refolding	0.013788
	snRNA processing	0.015794
MEsalmon	peripheral nervous system development	0.000392
	glial cell development	0.002722
	myelin assembly	0.002722
MEgreen	positive regulation of cell adhesion	1.51 × 10^−9^
	antigen processing and presentation of exogenous peptide antigen via MHC class II	9.66 × 10^−7^
	extracellular matrix organization	9.66 × 10^−7^
MEmidnightblue	defense response to virus	1.67 × 10^−25^
	response to virus	6.84 × 10^−24^
	antiviral innate immune response	8.36 × 10^−16^
MEyellow	aerobic respiration	2.17 × 10^−73^
	cellular respiration	3.31 × 10^−70^
	energy derivation by oxidation of organic compounds	2.21 × 10^−61^
MEturquoise	ribonucleoprotein complex biogenesis	2.24 × 10^−11^
	ribosome biogenesis	7.77 × 10^−8^
	rRNA metabolic process	1.41 × 10^−6^
MEcyan	regulation of circadian rhythm	0.000112
	rhythmic process	0.000112
	circadian rhythm	0.000133
MEmagenta	fatty acid oxidation	0.001356
	lipid oxidation	0.001356
	lipid modification	0.002489
MEblack	ribosome biogenesis	1.09 × 10^−33^
	ribonucleoprotein complex biogenesis	3.39 × 10^−33^
	ribosomal small subunit biogenesis	4.85 × 10^−33^

GO: Gene Ontology; ME: Module eigengene.

**Table 7:** Overlap between DEGs and Highly Connected Module Genes (kME > 0.8).

ME	Groups	Overlapping DEGs
MEpink	Sedentary	*Aldoc/Nrbp2/Slc25a30/Vwf/Prodh*
	Exercise	*Vwf/Prodh*
MEblue	Sedentary	*Spsb1/Maff/Pnpla2/Plim3/Galnt15*
	Exercise	*-*
MEbrown	Sedentary	*Lpl/Slc2a13/Ccdc8/Agpat2/Fgf1/Atp2a2/Smtnl1/Prox1/Ldhb/Acsf2/Rps6ka5/Dixdc1/Tpm3/Npr2/Tpd52l1/Tnnc1/Adamtsl4/Klhl34/Slc27a1/Kremen1/Tnni1/Nnt/Myl3/Gpam/Ift81/Decr1/Rerg/Eci1/Fgfrl1*
	Exercise	*-*
MEyellow	Sedentary	*Vegfa*
	Exercise	*-*
MEturquoise	Sedentary	*Ptpn3/Cep85l/Fbxo32*
	Exercise	*-*
MEmagenta	Sedentary	*Tns1/Rorc/Acad11/Golm1/Sdc4/Dgat2*
	Exercise	*-*

ME: Module eigengene; DEGs: differentially expressed genes.

## Data Availability

Transcriptome data can be found in GEO ID under accession number GSE298775 (https://www.ncbi.nlm.nih.gov/geo/query/acc.cgi?acc=GSE298775, accessed on 30 May 2025). Additional data in this review can be obtained upon request of the corresponding author.

## Supplementary Materials

The following supporting information can be downloaded at https://www.mdpi.com/article/10.3390/ijms26136525/s1.

## Abbreviations

The following abbreviations are used in this manuscript:

BPs	Biological Processes
CC	Cardiac cachexia
CC-Ex	Cardiac cachexia exercise group
CC-Sed	Cardiac cachexia sedentary group
DEG	Differentially expressed genes
Ex	Control exercise group
GO	Gene ontology
HF	Heart failure
KEGG	Kyoto Encyclopedia of Gene and Genomes
ME	Module eigengene
RV	Right ventricle
Sed	Control sedentary group
RIN	RNA integrity numbers
RNA-seq	RNA sequence
WGCNA	Weighted gene co-expression network analysis
